# Neutralization of the Pandemic Influenza A/H1N1 Virus with *Lama glama* Humanized Nanobodies (VHH)

**DOI:** 10.3390/antib14020042

**Published:** 2025-05-16

**Authors:** Zeila Yazmín Páez-Hernández, Jose Luis Stephano-Hornedo, Jose Alberto Bolaños-Prats, Iván Córdova-Guerrero, Mariana Macías-Alonso, Joaquín G. Marrero, Angel Pulido Capiz, Victor García González

**Affiliations:** 1 Facultad de Ciencias Químicas e Ingeniería, Universidad Autónoma de Baja California, Calzada Universidad 14418, Parque Industrial Internacional Tijuana, Tijuana 22390, Baja California, Mexico; zeila.yazmin.paez.hernandez@uabc.edu.mx (Z.Y.P.-H.); icordova@uabc.edu.mx (I.C.-G.); 2 Facultad de Ciencias, Universidad Autónoma de Baja California, Carretera Transpeninsular Ensenada-Tijuana No. 3917, Colonia Playitas, Ensenada 22860, Baja California, Mexico; stephano@uabc.edu.mx (J.L.S.-H.); jbolanos@uabc.edu.mx (J.A.B.-P.); 3 Instituto Politécnico Nacional, UPIIG, Av. Mineral de Valenciana, No. 200, Col. Fracc, Industrial Puerto Interior, Silao de la Victoria 36275, Guanajuato, Mexico; mmacias@ipn.mx (M.M.-A.); jgonzalezm@ipn.mx (J.G.M.); 4 Departamento de Bioquímica, Facultad de Medicina Mexicali, Universidad Autónoma de Baja California, Mexicali 21000, Baja California, Mexico; pulido.angel@uabc.edu.mx; 5 Centro de Innovación e Investigación en Salud (CIIS), Facultad de Medicina Mexicali, Universidad Autónoma de Baja California, Mexicali 21000, Baja California, Mexico; 6 Laboratorio Multidisciplinario de Estudios Metabólicos y Cáncer, Facultad de Medicina Mexicali, Universidad Autónoma de Baja California, Mexicali 21000, Baja California, Mexico

**Keywords:** nanobody, VHH, influenza, A/H1N1, phage display, neutralization

## Abstract

Background/Objetives: Nanobodies (VHH) have become an excellent tool for diagnosis, therapy, and research since VHH shows a high capability of recognizing and neutralizing antigens. VHHs are highly soluble and stable at high temperatures, and in the presence of chaotropic agents, they offer significant advantages over other biological therapeutic agents. This study aimed to identify and humanize VHH fragments with neutralizing potential against the influenza A/H1N1 virus. Methods: A library of VHH antibody fragments was produced by phage display technique against an inactivated influenza A/H1N1 vaccine. Three VHH sequences were selected and humanized. Specifically, the recognition capacity of the antibodies denominated 2-C10 and 2-C10H was confirmed by ELISA and western blot (WB), as well as their microneutralization capacity in a cellular model, suggesting their potential therapeutic use in patients infected with the influenza A/H1N1 virus. Molecular docking assays were used to support the mechanism of viral inhibition. Results: The VHHs 2-C10 and 2-C10H showed specific recognition of influenza A/H1N1 antigens by ELISA and Western Blot and demonstrated neutralizing activity in vitro. The optimal VHH, 2-C10H, showed 75% neutralization capacity at a concentration of 1.56 μg/mL against the A/H1N1 viral strain, potentially through the inactivation of hemagglutinin protein, a phenomenon supported by molecular docking assays. Conclusions: This study presents a strategic approach to identify VHH candidates that may be useful for diagnosing and potentially treating patients already infected by the A/H1N1 virus, as it may reduce the severity of their symptoms.

## 1. Introduction

Influenza is an acute respiratory disease caused by viruses from the *Orthomyxoviridae* family, specifically genera A, B, and C. Types A and B represent the most significant concerns in terms of epidemiology and public health due to their potential for zoonotic transmission. Both types are highly contagious and responsible for annual epidemics that affect a large portion of the population, often leading to severe illness or death, especially in vulnerable groups [1].

Influenza A viruses are further classified into subtypes based on the antigenic properties of their envelope glycoproteins: hemagglutinin (HA) and neuraminidase (NA). HA facilitates viral entry by binding to α2,3-linked sialic acid receptors on host cells and promoting membrane fusion. NA assists in releasing new viral particles by cleaving terminal sialic acids from host glycoproteins and gangliosides, thereby enhancing viral spread. These proteins are essential targets for diagnostic methods and antiviral therapies [2,3].

The first pandemic of the 21st century was caused by the Influenza A/H1N1 strain, which spread to 214 countries and caused over 18,449 deaths worldwide. Although incidence has declined since its emergence, sporadic cases have been reported globally since 2018, underscoring a persistent threat [1].

Vaccination remains the most effective strategy to prevent influenza and is updated annually to mitigate epidemic outbreaks [4]. In addition, antiviral drugs such as adamantanes (e.g., rimantadine) and neuraminidase inhibitors (e.g., zanamivir, oseltamivir, peramivir) are used to treat infections. However, viral resistance to these drugs is a growing concern, contributing to increased morbidity, mortality, and economic burden [5,6,7,8].

An alternative approach is passive immunotherapy using anti-influenza antibodies. Immunoglobulin A (IgA) and G (IgG), the predominant isotypes in mucosal tissues, can recognize and neutralize the virus or prevent its binding to host cells [9,10,11]. Consequently, several monoclonal antibodies (mAbs) have been developed and evaluated against various influenza subtypes, including A/H1N1pdm09 [2,12,13,14,15].

Heavy chain-only antibodies (HCAbs) have emerged as promising alternatives in recent years. These antibodies consist solely of two heavy chains, each containing constant domains CH2 and CH3, but lacking the CH1 domain and light chains [16]. The antigen-binding region of HCAbs, known as the VHH domain or nanobody, comprises a single variable domain derived from the heavy chain. Nanobodies are the most minor naturally occurring antigen-binding fragments (12–15 kDa) and can target cryptic or concave epitopes often inaccessible to conventional antibodies [17,18,19,20].

VHH nanobodies offer advantages over traditional mAbs, including comparable or superior specificity and affinity, enhanced hydrophilicity and solubility, and improved tissue penetration. Their simple structure allows for the design of multivalent constructs and expression in scalable production systems such as *Escherichia coli*, *Pichia pastoris*, and *Saccharomyces cerevisiae* [21,22,23].

Despite their many advantages and a lower propensity to induce anti-drug antibodies (ADAs), VHHs derived from non-human sources may still trigger immune responses in humans. Humanization strategies are employed to mitigate this immunogenicity. These involve grafting the original nanobody’s complementarity-determining regions (CDRs) onto human antibody framework regions that exhibit high sequence homology. This technique aims to retain the nanobody’s antigen-binding specificity and therapeutic efficacy while reducing the likelihood of recognition by the human immune system [24,25,26,27].

This study aimed to investigate the potential of VHH nanobodies as antiviral agents against the influenza A/H1N1 virus. A nanobody library was generated using phage display technology from animals immunized with a monovalent A/H1N1 vaccine. From 80 sequenced clones, eight unique sequences were identified, and three (1-C7, 1-D8, and 2-C10) were selected for further characterization. These VHHs were humanized and evaluated using ELISA and Western blot assays with a trivalent vaccine antigen. Two humanized clones, 1-C7H and 2-C10H, were then assessed in viral neutralization assays. Results showed that 2-C10H effectively inhibited A/H1N1, likely through inactivation of the HA protein, as supported by molecular docking analyses.

## 2. Results

### 2.1. Generation of a Diverse VHH Library Against Influenza A/H1N1 Post Llama Vaccination

Immunization of llamas with four doses of the monovalent A/California/7/2009 (A/H1N1) vaccine (150 μg per dose) induced a strong humoral response, as evidenced by elevated antibody titers. Peripheral blood lymphocytes collected post-immunization enabled the construction of a highly diverse VHH phage display library. Following amplification and cloning of VHH fragments into the pCOMB3X vector, transformation into *E. coli* XL1-Blue achieved efficient surface display of nanobodies. The resulting library exhibited substantial diversity, providing a robust platform for the downstream selection of high-affinity antigen-specific VHH clones.

### 2.2. Identification of High-Affinity Nanobodies and Diverse Nanobody Candidates

Three rounds of biopanning against immobilized trivalent A/H1N1 Vaxigrip^®^ vaccine antigens enriched for nanobody candidates with strong binding activity. Screening identified 80 phage clones demonstrating apparent antigen binding. Subsequent sequence analysis revealed eight unique VHH sequences, reflecting underlying library diversity. Among these, three candidates designated 1-C7, 1-D8, and 2-C10 that were selected based on their distinct sequence profiles and superior binding performance, establishing a focused panel for further functional characterization.

### 2.3. Humanization Preserves Structural Integrity and Solubility of Selected VHHs Structural and Solubility Integrity of Humanized Nanobodies

**Figure 1:**
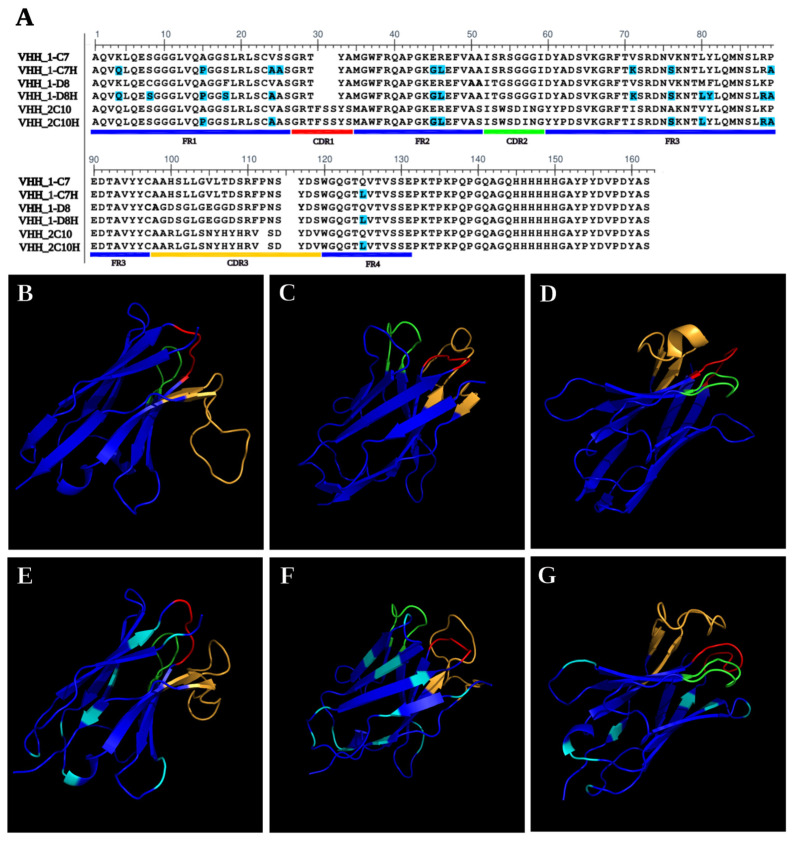
(**A**) Sequence alignment and 3D representation of (**B**) 1-C7, (**C**) 1-D8, (**D**) 2-C10, (**E**) 1-C7H, (**F**) 1-D8H and (**G**) 2-C10H VHH. Protein sequence alignments translated from individual DNA sequence clones obtained by phage display targeting AH1N1 2009 pdm. The Framework regions (FRs) and Complementary-determining regions (CDRs) are highlighted in the alignment, with sequence modification shown in cyan. Each native VHH is shown alongside its humanized version. 3D representations of each native VHH are compared with its humanized version; CDRs are color-coded as red for CDR1, green for CDR2, and yellow for CDR3; and FRs are colored royal blue.

The selected VHHs (1-C7, 1-D8, and 2-C10) underwent humanization via CDR grafting to reduce immunogenicity and enhance therapeutic applicability. Each humanized nanobody, designated 1-C7H, 1-D8H, and 2-C10H, retained the original CDR regions while incorporating 9, 10, and 14 amino acid substitutions (highlighted in cyan) in clones 1-C7, 1-D8, and 2-C10, respectively within the framework regions as is shown in Figure 1A. In silico solubility predictions using Protein-sol (https://protein-sol.manchester.ac.uk/ accessed on 29 December 2022) confirmed that the modifications did not significantly impact protein solubility. Structural modeling with Phyre2 demonstrated that both native and humanized nanobodies preserved the characteristic β-strand-rich fold topology (Figure 1B–G). The CDR regions remained conformationally similar between native and humanized forms, with sequence modifications localized outside the antigen-binding sites. These results highlight the structural robustness and solubility retention of the humanized VHHs, supporting their suitability for downstream expression, purification, and functional validation [28].

### 2.4. Validation of Recombinant VHH Production and Purification Efficacy

Following the humanization process, the VHH-encoding genes were subcloned from the pUC57 vector into the expression vector pCOMB3X and transformed into *Escherichia coli* TOP10F cells for recombinant production. The constructs were verified by restriction enzyme digestion with Sfi1, confirming correct insertion. The sequence comparison of VHH and the humanized counterparts are shown in Figure 1A.

**Figure 2:**
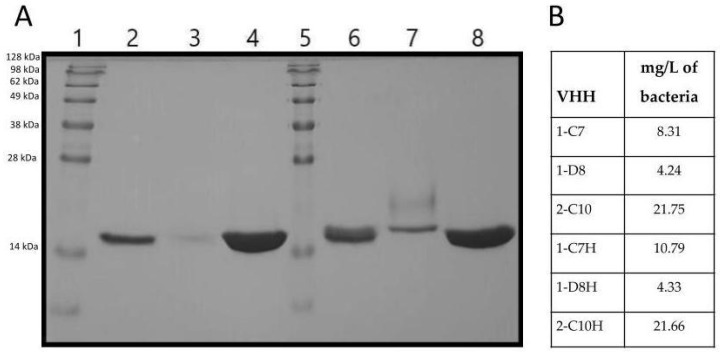
(**A**): SDS-PAGE analysis of VHH 1-C7, 1-D8, 2-C10, 1-C7H, 1-D8H and 2C-10H under non-reducing conditions. A 12% gel was stained. Lane 1: MPM SeeBlue^®^ plus2 prestained standard. Lane 2: 1-C7 VHH (15.63 kDa). Lane 3: 1-D8 VHH (15.94 kDa). Lane 4: 2-C10 VHH (16.1 kDa). Lane 5: MPM SeeBlue ^®^ plus2 prestained standard. Lane 6: 1-C7H VHH (15.46 kDa). Lane 7: 1-D8H VHH (15.31 kDa). Lane 8: 2-C10H VHH (16.13 kDa). (**B**): Yield of purified VHH mg/L bacteria culture.

Additionally, the six VHH variants (1-C7, 1-D8, 2-C10, 1,C7H, 1-D8H and 2-C10H) were expressed and purified using nickel-affinity chromatography. SDS-PAGE analysis showed bands corresponding to ~17 kDa, consistent with the expected size of VHHs, confirming the production of full-length VHH proteins (Figure 2).

Among all constructs, the highest expression levels and purification yield were obtained from clones 2-C10 and 2-C10H, which also showed superior stability post-purification (Figure 2). These characteristics support their suitability for downstream functional assays and reinforce their potential for scalable production under therapeutic applications.

### 2.5. Differential Antigen Recognition Profiles of Native and Humanized VHHs

**Figure 3:**
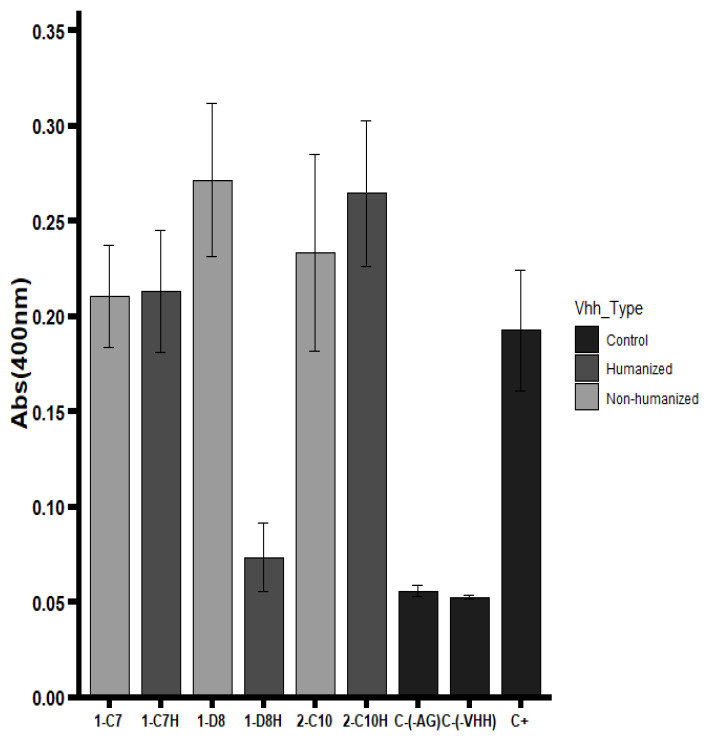
Recognition ELISA assay. The trivalent Vaxigrip^®^ vaccine was used as the antigen. Two negative controls were included in the experiment: (C-(-AG)) and (C-(-VHH)), without the antigen (Ag) and without VHH, respectively.

Binding activity of native and humanized VHHs was evaluated by ELISA using the trivalent Vaxigrip^®^ vaccine as the antigen. The humanized VHH 2-C10H demonstrated full retention of antigen recognition, performing equivalently to its native counterpart 2-C10 (Figure 3). Similarly, 1-C7H maintained binding capacity comparable to the native 1-C7, indicating that the humanization strategy successfully preserved the structural integrity of the antigen-binding sites.

Conversely, the 1-D8H variant exhibited a complete loss of binding activity. Although the complementary-determining regions (CDRs) remained unchanged, extensive amino acid substitutions within the framework regions (FRs) likely disrupted proper folding or altered critical residues involved in antigen interaction [29,30]. This highlights the sensitivity of certain scaffolds to framework modifications and the critical balance needed between reducing immunogenicity and maintaining structural fidelity.

To further characterize antigen specificity, complementary ELISA assays were performed using viral protein preparations from A/H1N1. These analyses revealed that clone 2-C10 specifically recognized hemagglutinin (HA), a ~62 kDa glycoprotein that constitutes the major antigenic component of the viral envelope. In contrast, clones 1-C7 and 1-D8 predominantly bound to a distinct epitope, likely corresponding to neuraminidase (NA), the second most abundant surface glycoprotein on the influenza virus [31].

Overall, the results indicate that humanization can maintain antigen recognition in most cases, though scaffold-specific factors may influence the extent of preservation.

### 2.6. Confirmation of Antigen Specificity for 2-C10H via Western Blot Analysis

**Figure 4:**
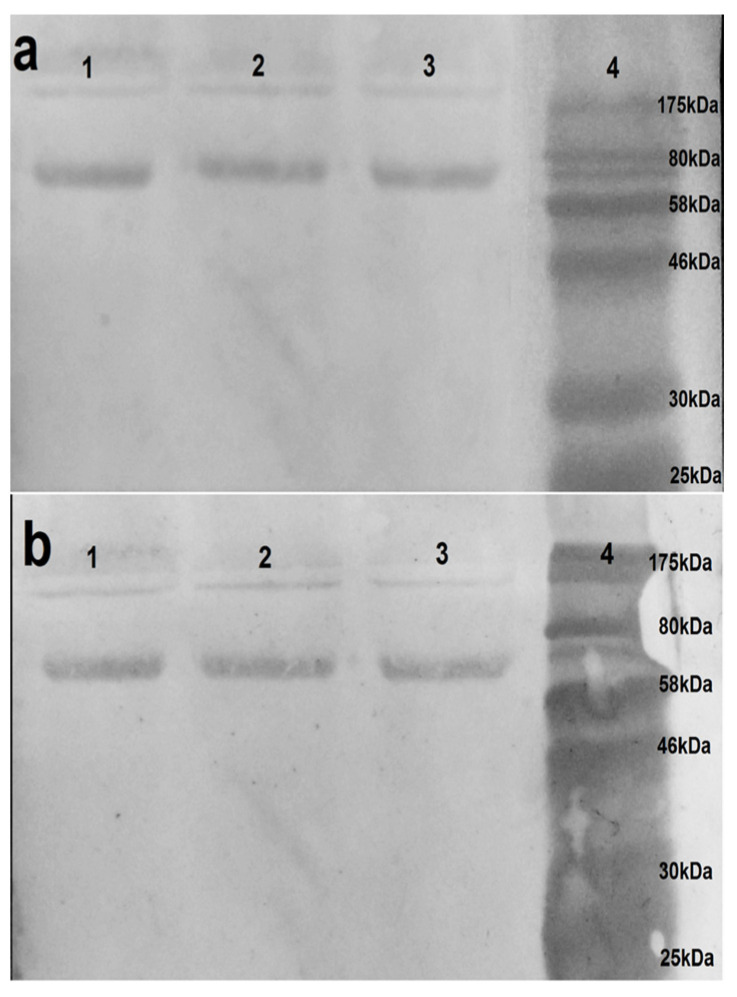
Western blot analysis of (**a**) 2-C10 and (**b**) 2-C10H VHH against the Vaxigrip^®^ vaccine. Results are shown in triplicate (lanes 1, 2 and 3).

Western blot assays were performed to confirm the specificity of VHH binding observed in ELISA and to evaluate the ability of the VHHs to recognize native viral proteins. Under non-reducing conditions, VHH 2-C10H identified a distinctive protein band between 61–63 kDa, consistent with the expected molecular weight of hemagglutinin (62 kDa) (Figure 4), thereby confirming its specific recognition of this glycoprotein. In contrast, neither the native nor humanized clones 1-C7 and 1-D8 produce detectable signals under the same conditions. The absence of binding by 1-C7 and 1-D8 variants may reflect epitope masking or structural dependency, potentially associated with recognition of conformational epitopes within higher-order protein complexes, such as oligomeric neuraminidase, which may not resolve efficiently under SDS-PAGE conditions.

Overall, these findings corroborate the ELISA results and support the continued characterization of 2-C10H as a promising candidate for further functional and antiviral analyses.

### 2.7. Neutralization Profiles of Native and Humanized VHHs Against Influenza Strainshumanize

**Figure 5:**
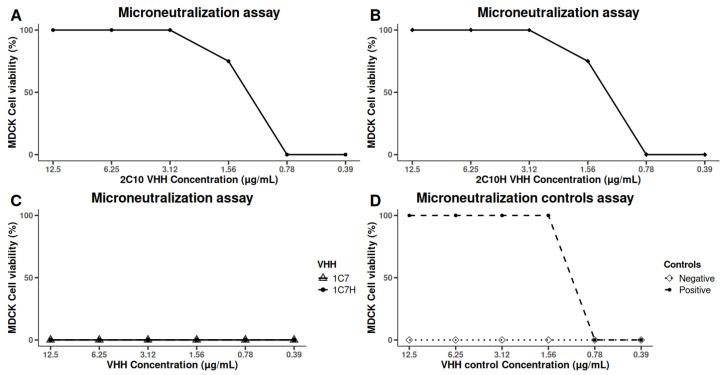
A/H1N1 neutralization assays in MDCK cells using the A/H1N1/California/07/2009 strain. (**A**) VHH 2-C10, (**B**) 2-C10H, (**C**) 1-C7, 1-C7H, (**D**) Controls. A neutralizing mouse serum was used as a positive control, and a non-neutralizing antibody was used as a negative control. All assays were performed in quadruplicate.

The neutralizing capacity of the humanized VHHs was assessed using microneutralization assays in MDCK cells overexpressing the α2,6-sialyltransferase I (ST6Gal I) receptor, enhancing their susceptibility to A/H1N1 infection. Consistent with previous observations, the native VHH 2-C10 demonstrated potent neutralizing activity against the A/California/07/2009 (H1N1) strain. Importantly, the humanized variant 2-C10H retained comparable antiviral efficacy, achieving 100% inhibition of cytopathic effects at 3.125 μg/mL and 75% inhibition at 1.56 μg/mL (Figure 5).

In contrast, neither the native nor humanized forms of 1-C7 exhibited detectable neutralizing activity against this strain. Furthermore, no neutralizing effects were observed against heterologous influenza strains A/H3N2 Panama (A/Panama/7/2004) or A/H1N1 Brisbane (A/Brisbane/59/2007) by any of the tested VHHs.

These findings highlight the strain-specific neutralizing potential of 2-C10 and its humanized counterpart 2-C10H and are consistent with the antigen recognition patterns observed in previous ELISA and Western blot assays, indicating limited cross-reactivity with other influenza subtypes.

### 2.8. 2-C10H Binds Specifically to Influenza Hemagglutinin in Molecular Docking Analysis

**Figure 6:**
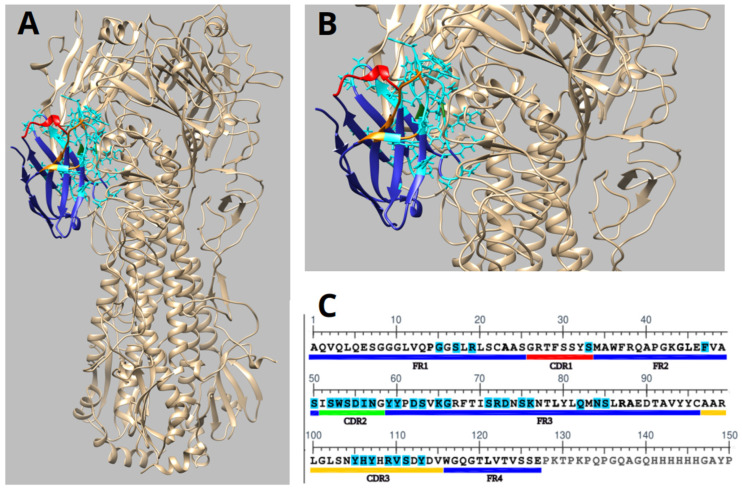
Characterization of 2-C10H and hemagglutinin (PDB ID: 3LZG) through molecular docking. (**A**) Docking of hemagglutinin protein showed in beige and 2-C10H in blue. For 2-C10H, CDR1 is identified red, CDR2 green, and CDR3 orange. (**B**) Image zoom identified the interaction residues of 2-C10H as cyan. (**C**) 2-C10H sequence, highlights in cyan the interaction residues (Arg^109^, Arg^19^, Arg^72^, Asn^57^, Asn^84^, Asp^55^, Asp^62^, Asp^73^, Gln^82^, Gly^15^, Gly^66^, His^106^, Ile^56^, Lys^65^, Lys^76^, Phe^47^, Ser^111^, Ser^17^, Ser^33^, Ser^50^, Ser^52^, Ser^54^, Ser^63^, Ser^71^, Ser^75^, Ser^85^, Trp^53^, Tyr^105^, Tyr^107^, Tyr^113^, Tyr^59^, Tyr^60^, Val^110^).

Molecular docking analysis demonstrated that the humanized nanobody 2-C10H specifically recognizes the 009 H1N1 influenza virus hemagglutinin (HA) (PDB:3LZG), a major viral surface glycoprotein involved in host cell entry. The molecular docking experiments were modelled using the monomeric form of hemagglutinin, revealing a stable binding interface (Figure 6A,B).

The structural representation showed that the CDR2 and CDR3 contribute most significantly to antigen recognition as they contain most of the contact residues involved in binding. These findings are consistent with the expected role of CDR loops in mediating high-affinity antigen recognition (Figure 6B,C).

Therefore, molecular docking of 2-C10H with hemagglutinin indicated a strong interaction, with an S-score of −92.71 for the monomer. The S-score, reflecting predicted binding energy in MOE, with lower values indicating higher affinity, supports the biological evidence of 2-C10H recognition of A/H1N1 via the hemagglutinin surface.

Based on our molecular docking data, 2-C10H interacts with several surface-exposed residues on the hemagglutinin globular head domain. Notably, key contact residues on HA include Lys68, Asp171, and Ile269, which lie in close proximity (≤4 Å) to residues Lys65, Lys76, and Tyr59 of 2-C10H. These residues are located near the receptor-binding site (RBS), although they do not directly overlap with the sialic acid-binding pocket (Figure 6A–C).

The spatial arrangement of these interactions strongly suggests a steric blockade, whereby 2-C10H partially occludes the RBS or obstructs the approach of host sialic acid receptors. This would prevent the viral attachment process without requiring direct binding to catalytic or fusogenic motifs.

## 3. Discussion

Severe influenza infections occur every season, particularly among high-risk populations such as children, the elderly, pregnant women, and immunocompromised individuals. In these groups, vaccines and antiviral drugs often provide suboptimal protection. Therefore, passive immunization using antibodies has emerged as a promising alternative to conventional antiviral therapies.

Since the FDA approved the first monoclonal antibody in 1986 [32], therapeutic mAbs have expanded significantly and now represent a major class of antiviral agents under development. These antibodies have been successfully directed against a wide range of viruses, including human immunodeficiency virus (HIV-1), influenza viruses, hepatitis C virus (HCV), respiratory syncytial virus (RSV), enteric viruses, and coronaviruses [33].

Nanobodies, derived from the variable domain of camelid heavy-chain-only antibodies, offer notable therapeutic advantages as they can interfere with different stages of the viral replication cycle [34]. For instance, nanobodies such as 2-C10H can bind to specific epitopes on hemagglutinin, thereby preventing the virus from attaching to host cells. In addition, VHH antibodies exhibit favorable properties, including low immunogenicity and high stability across a broad range of environmental conditions, making them an attractive candidate for antiviral therapy [35].

Their small size (~15 kDa) allows access to hidden or conformational epitopes often inaccessible to full-sized antibodies [36], enhancing their potential as therapeutic and diagnostic tools. Furthermore, VHHs demonstrate exceptional thermal and chemical stability, which supports their efficient recombinant production and broad applicability across experimental and clinical platforms [20].

In this study, we successfully generated and characterized VHH nanobodies targeting the influenza A/H1N1 California/07/2009 vaccine. Using phage display technology, we identified three nanobodies (1-C7, 1-D8, and 2-C10) with specific binding capabilities. A humanization strategy was applied to improve compatibility with the human immune system and reduce immunogenicity while maintaining their affinity and stability to enhance their potential for clinical applications.

Humanization process was performed through complementarity-determining region (CDR) grafting, which led to a limited number of amino acid substitutions in the framework regions (FRs): 9 in 1-C7H, 10 in 1-D8H, and 14 in 2-C10H. Structural modeling suggested that the characteristic β-strand-rich topology of the nanobodies was preserved, supporting retention of conformational integrity (Figure 1). This was further confirmed experimentally by the ability of 2-C10H to recognize hemagglutinin under native conditions (Figure 4 and Figure 6).

Expression and purification of all VHHs revealed a molecular weight of ~17 kDa, as expected. The highest production levels were achieved with 2-C10 and 2-C10H, indicating their potential for large-scale manufacturing. Lower yields observed for 1-D8 and 1-D8H may reflect tag accessibility issues rather than intrinsic expression defects. Functional assays demonstrated that five of six VHHs retained antigen binding, with the exception of 1-D8H, which likely lost function due to structural perturbations following humanization [37,38].

The western blot analysis confirmed the target specificity of 2-C10H, which successfully recognized hemagglutinin (HA) at its expected molecular weight (62 kDa). In contrast, 1-C7H and 1-D8H showed no detectable signals under the same conditions, possibly reflecting recognition of conformational epitopes disrupted during SDS-PAGE or epitopes located within oligomeric complexes such as neuraminidase (NA).

Importantly, microneutralization assays demonstrated that both native 2-C10 and its humanized variant 2-C10H (Figure 5) effectively neutralized the A/California/07/2009 (H1N1) strain at low concentrations, whereas 1-C7 and 1-C7H lacked neutralizing activity. No neutralization was observed against a/H3N2 Panama and A/H1N1 Brisbane strains, suggesting strain-specific recognition. This finding is consistent with previous reports by Voronina et al. (2021), who reported that VHHs could exhibit potent activity against single strains but limited cross-reactivity, highlighting the challenge of developing broadly neutralizing nanobodies [39].

Previous studies have implemented similar strategies for generating humanized nanobodies against influenza. For example, Barbieri et al. (2024) reported a panel of humanized anti-HA nanobodies generated via CDR grafting that retained functional binding and exhibited improved pharmacokinetics in animal models. Similarly, Detalle et al. (2016) developed a VHH therapeutic against RSV, which demonstrated potent neutralizing activity and good tolerance after inhaled delivery in clinical trials. These studies support the feasibility of translating VHH-based strategies into clinical applications [40,41].

Compared to these previous studies, our work provides a detailed integration of structural modeling, humanization, and functional validation. Notably, our study directly examines the impact of humanization on both structure and function, highlighting that while 2-C10H retained full functional activity post-humanization, 1-D8H suffered loss of binding likely due to framework alterations. Furthermore, 2-C10H is a promising candidate due to its high yield, retained binding, and neutralizing activity post-humanization, characteristics essential for preclinical development.

Currently, VHHs have demonstrated clinical success across various fields. Caplacizumab, a VHH targeting von Willebrand factor, was the first nanobody approved for human use [42]. In the context of infectious diseases, VHHs have been widely explored in the fight against SARS-CoV-2 by targeting the virus’s Spike protein or by creating conjugates of the neuraminidase inhibitor zanamivir attached to a VHH that recognizes the kappa light chains of mouse immunoglobulins [43,44]. In oncology, VHHs have been investigated for the treatment of tumors that overexpress HER2 and EGFR since their small size facilitates better tumor penetration and biodistribution in preclinical phases regard monoclonal antibodies; in autoimmune diseases, VHHs have been designed to target inflammatory cytokines such as TNF-**α** and IL-6 [45]. Future studies could optimize its affinity and evaluate its efficacy in In vivo preclinical models, opening the way for its application in innovative antiviral strategies. However, besides the wide range of applications of VHHs, further studies are still required to validate their safety in various clinical fields.

## 4. Materials and Methods

### 4.1. Immunization Protocol

A 10 years old male llama was immunized intramuscular (IM) 4 times with 150 μg of monovalent vaccine antigen (A/California/7/2009(H1N1)-Sanofi Pasteur (Birmex) 10X concentrated, using aluminum hydroxide as adjuvant at two weeks’ interval (Salud S. Cofepris. Datasheet Vaxigrip), seven days after the last immunization ~500 mL of blood were collected in 100 mM EDTA by jugular puncture. According to the ethics statement (Section 4.11).

### 4.2. Construction of Phage Display Library

Peripheral blood lymphocytes were extracted following the Lymphoprep protocol. Total RNA extraction was carried out with the RNA Stat reagent following the manufacturer’s instructions, quantity and purity were verified in Nanodrop. cDNA was synthesized by reverse transcriptase from total RNA with the SuperScript-III kit (Invitrogen, Co., Waltham, MA, USA) following the manufacturer’s instructions.

The variable domains of single heavy-chain antibodies (VHH) were amplified using the specific primers for short (VH1Back-SfiI and Lam7-SfiI) and for long-chain fragments (VH1Back-SfiI and Lam8-SfiI). In addition, the PCR product (PPCR) was washed and quantified.

The immune library was obtained as follows: the PCR products were purified by agarose gel electrophoresis, digested with Sfi I, and ligated to the linearized pCOMB3X phagemid vector. The resulting VHH library was cloned into *Escherichia coli*, then infected M13 helper phage to express the VHHs as pIII fusion protein on the filamentous phage surface, cultured with the corresponding antibiotics (CB 50 μg/mL, TC 30 ug/mL), and harvested by polyethylene glycol (PEG) precipitation. Briefly, the re-amplified phages in the supernatant were precipitated with 8 g of PEG-8000 and 6 g of NaCl and then incubated on ice for 30 min. Next, the phages were pelletized at 15,000× *g* for 15 min at 4 °C. Finally, the supernatant was discarded, and the pellet was resuspended in 2 mL of 1% BSA-PBS and filtered through a 0.22 μm membrane.

### 4.3. Biopanning and Selection of A/H1N1 Potential Binders

Phage display was performed according to Barbas et al., 2021 [46]. Three panning rounds against 300 ng/well of monovalent vaccine A/H155N1 2009 were performed in 96-well plates. All the rounds were vigorously washed with TBS-Tween 20 0.2% (5, 10, and 15 times for 1st, 2nd, and 3rd rounds, respectively). TOP10F’ cells were infected with the last round of phages for individual search by soluble infection. The individual clones were placed in two 96-well plates and grown ON in SB medium with CB (50 ug/mL).

The next day each clone was inoculated into two 96-well plates in fresh medium and brought to an optical density of 0.5–0.6 at 600 nm, and expression was induced with IPTG 1 mM. The supernatants containing the individual VHHs were tested in the Elisa assay. Briefly, the plate was coated with monovalent A/H1N1 vaccine overnight, washed with H_2_O and blocked with PBS-5% milk (BD-Difco) for 2 h at 37 °C. The blocker solution was discarded, and the supernatant of each expressed clone was mixed 1:1 PBS-Milk 5% and incubated 2 h at 37 °C under the arrangement designed for the plate; VHHs were discarded, and the plate was washed (10 times) in H_2_O, the secondary antibody 6-His-HRP (BETHYL, Cat. A190-114P) were added (1:1000) and incubated 1 h at 37 °C. Finally, the antibody was discarded and each well was washed 10 times with H_2_O. TMB substrate solution (peroxidase substrate, (AGDIA, Cat. ACC 00412/0060) was added, and the plates were read at 405 nm intervals of 15 and 30 min. Plasmid DNA from recognition clones was sequenced. Three of these clones were selected for the following assays due to their high recognition capacity against the pandemic A/H1N1 virus.

### 4.4. Humanization Process

The 1-C7, 1-D8, and 2-C10 clones were subjected to the humanization process achieved by CDR grafting based on framework regions homology (FWR). The 3D design of the humanized nanobodies was modeled with Swiss-PdbViewer 4.1.0 program, modifying the relative position of specific residues of the nanobody examining with the ProteinSol program to avoid solubility reduction of the humanized VHHs. The 3EAK antibody of the ECSB Protein database Data Ink was used as a scaffold.

The synthesis of the gene sequences encoding the humanized antibodies was performed by the Genscript Company (Piscataway, NJ, USA). The corresponding VHH humanized DNA fragments were inserted in the linearized pCOMB3X (Addgene, Cat. #63890) vector using the endonuclease SfiI (NEB, Cat. R0123S) sites and chemo transformed into *E. coli* Top10F’.

### 4.5. Selected VHH Expression and Purification

The VHH was purified through nickel affinity, a clone was placed in 5 mL of SB medium with 50 ug/mL CB, incubated at 37 °C and 250 rpm ON. The next day, 2.5 mL of culture medium was added to 250 mL of fresh medium induced with IPTG 1 mM and incubated at 300 rpm 20 h at 37 °C. The culture media were centrifuged at 4000 rpm for 20 min at 4 °C, and the pellet was discarded. Ni-NTA agarose beads (QIAGEN, Hilden, Germany, Cat. 30210) were added to the VHH medium and incubated at 4 °C ON in the rotating platform. The beads were harvested by centrifugation at 1000 rpm for 1 min, transferred to chromatography columns (Bio-Rad Laboratories, Hercules, CA, USA), and washed thrice with washing buffer (50 mM NaPO4, 20 mM imidazole, 0.3 M NaCl, pH 8.0), later VHH was eluted with elution buffer (50 mM NaPO4, 300 mM imidazole, 0.3 M NaCl, pH 8.0). The purification was verified with SDS-PAGE and quantified with nanodrop spectrophotometer (Thermo Fisher Scientific, Waltham, MA, USA). The purified protein for the microneutralization assay was filtered through a 0.2 μm filter (Millipore Sigma, Burlington, MA, USA).

### 4.6. ELISA Assay

96-well plates were coated overnight at 4 °C with 50 μL of trivalent Vaxigrip vaccine and blocked with 5% BSA-PBS for 2 h at 37 °C. After washing three times with PBS 1X PBS-0.05% Tween (PBST), 50 μL of the corresponding VHH were added and incubated overnight. After washing three times with 5% BSA-PBS, 50 μL of a 6-His-HRP antibody diluted 1:10,000 in 1% BSA-1X PBS was added and incubated 1 h at 37 °C, washed three times with PBST and twice with H_2_O. The plate was revealed with p-TMB in microplate reader Multiskan GO (Thermo Scientific, Waltham, MA, USA) at 405 nm after 15 min of darkness.

### 4.7. VHH Recognition Assay Against Trivalent Flu Vaccine by Western Blot

Electrophoresis was performed under native conditions of the vaccine concentrated 10 times on the Centricon YM-30 column (Millipore, Cat. #42410). Proteins were transferred from the gel to a nitrocellulose membrane and blocked with 5% PBS-M ON at 4 °C. The blocker was discarded, and 1 ug/mL VHH was added for incubation at 12 h at 4 °C with shaking. The VHH was discarded and washed thrice with PBS-T 0.05%. 6-His-HRP antibody 1:1000 PBS was added and incubated 1 h at 37 °C with shaking. The residual antibody was discarded and washed three times with PBS-T and twice with PBS. The WB was revealed with TMB.

### 4.8. Propagation of Influenza Virus and Determination of the Infective Dose Title (TCID_50_)

Three strains of the virus were used: the pandemic A/H1N1 California (A/California/07/2009), the A/H3N2 Panama (A/Panama/7/2004), and the A/H1N1 Brisbane (A/Brisbane/59/2007), these strains of the virus were duplicated in Madin-Darby Canine Kidney cells (MDCK α2.6). The infective dose of tissue culture 50% (TCID50) for each virus was determined by titration of viruses diluted serially in MDCK α2.6 cells and calculated by the Reed and Muench method [47]. Briefly, 96-well plates were seeded with MDCK α2.6 cells in MEM medium and incubated for three days at 37 °C and 5% CO_2_ until 80–90% confluence. The MEM medium was then discarded, and the wells were washed with PBS. Each well received 180 μL of medium without FBS. For virus titration, a 1:10 initial dilution was prepared by adding 20 μL of virus to the first well. Serial 10-fold dilutions were performed by transferring 20 μL from one well to the next across 11 wells discarding the last 20 μL of the last well. The 12th in each row, containing only medium without virus, served as a control.

### 4.9. Microneutralization Assays in MDCK α2,6 Cells

The MDCK α2,6 monolayer cells were treated with 100 TCID50 per 50 µL of the virus, pre-incubated with serial dilutions of the VHH 1-C7, 1-C7H, 2-C10, and 2-C10H. A neutralizing mouse serum was used as a positive control, and a non-neutralizing VHH antibody as a negative control. For the microneutralization assay, 96-well culture plates were seeded with MDCK α2,6 cells in MEM/FBS medium with Pur 10 mg/mL, incubated at 37 °C and 5% CO_2_ for 3 days. VHH dilutions were prepared starting from an initial concentration of 200 ng/μL. Briefly, 125 μg of diluent (plain MEM) were added to wells 1–11 of each row. Then 125 μL of VHH dilution were added to well 1 and serial twofold diluted across the row by transferring 125 μL from one well to the next, discarding the final 125 μL from well 11.

Each virus was diluted to a concentration of 100 TCID50 in MEM, and 125 μL of the virtual suspension were added to wells 1–11 containing the VHH dilutions. The virus-VHH mixtures were incubated 1 h at 37 °C in 5% CO_2_ to allow neutralization.

The microneutralization assay was carried out for quadruplicate (four replicate wells per dilution). After removing the culture medium and washing the cell monolayers once with PBS, 150 μL of MEM/HEPES supplemented with TPCK were added to wells 1 to 11 of the same row. Well 12 received 200 μL of medium only and served as a cell control. After incubation of the Virus-VHH reaction, 50 μL of each mixture was added to the corresponding four rows. The plates were incubated at 37 °C and 5% CO_2_, and cytopathic effect was read manually by bright field microscopy on day 4 post-infection.

### 4.10. Docking Experimentation

This strategy was based on the characterization of structure of the hemagglutinin from the A/California/04/2009 H1N1 (Pdb code: 3LZG) and 2-C10 and 2-C10H, considering a previous strategy of our group [48].

The structures of ligand molecules were obtained from the PubChem database (21), Cry (CID 160254), and fulvestrant (CID 104741). The PDB three-dimensional structure of ERα at 1.9 Å resolution (3ERT) and HER2+ at 2.25 Å resolution (3PP0) were characterized. The protein structures were prepared by removing water and small molecules, leaving only the protein structure. Ligand and receptor were 3D-protonated, and energy minimization was done using Molecular Operating Environment (MOE) software 2022.02 with default parameters under the AMBER99 force field. The ligand generates different conformations using a stochastic search in MOE default parameters.

Molecular docking was set as the default parameter for MOE software, and the pre-conformations were employed. To analyze docking results, MOE identifies salt bridges, hydrogen bonds, hydrophobic interactions, sulfur-LP, cation-π, solvent exposure and gives the S score (MOE). Ligand interactions with target proteins were predicted based on the S score.

### 4.11. Institutional Review Board Statement

The study was approved by the Ethics Committee and Evaluation of Facultad de Ciencias, Universidad Autónoma de Baja California (Protocol CEEIP/23-12-02).

## 5. Conclusions

In this study, we generated an immune library from llama (*Lama glama*) immunized with the A/H1N1 California 2009 trivalent VAXIGRIP^®^ vaccine and successfully selected three potential binders: 2-C10, 1-C7, and 1-D8. These nanobodies were subsequently humanized, yielding the 2-C10H, 1-C7H, and 1-D8H variants. Among them, 2-C10H and 1-C7H retained the ability to recognize the vaccine’s protein antigens; and 2-C10H demonstrated the capacity to neutralize influenza virus in MDCK cells. Notably, 2-C10H preserved solubility, antigen recognition, and neutralization capacity comparable to its parental nanobody, 2-C10. These findings position 2-C10H as a promising candidate for further preclinical evaluation, including testing in more physiologically relevant models such as larger mammals. Therefore, the experimental strategy based on *Lama glama* immunisation coupled with phage display technology, provides a robust and adaptable platform for development of novel biotherapeutics. This approach holds significant potential not only for the rapid response to emerging infectious diseases but also for the development of targeted treatments for chronic and degenerative diseases.

## Data Availability

Data are contained within the article.
